# DIAGNOSIS OF ENDOCRINE DISEASE: Thyroglobulin measurement using highly sensitive assays in patients with differentiated thyroid cancer: a clinical position paper

**DOI:** 10.1530/EJE-14-0148

**Published:** 2014-08

**Authors:** Luca Giovanella, Penelope M Clark, Luca Chiovato, Leonidas Duntas, Rossella Elisei, Ulla Feldt-Rasmussen, Laurence Leenhardt, Markus Luster, Camilla Schalin-Jäntti, Matthias Schott, Ettore Seregni, Herald Rimmele, Jan Smit, Frederik A Verburg

**Affiliations:** 1 Department of Nuclear Medicine PET Centre and Thyroid Unit, Oncology Institute of Southern Switzerland Via Ospedale 12, Bellinzona, 6500 Switzerland; 2 Clinical Laboratory Services Queen Elizabeth Hospital Birmingham Birmingham UK; 3 Department of Internal Medicine and Endocrinology Fondazione Salvatore Maugeri IRCCS, University of Pavia Hospital 27100, Pavia Italy; 4 Endocrine Unit Evgenidion Hospital, University of Athens Medical School Athens Greece; 5 Department of Endocrinology University Hospital Pisa Pisa Italy; 6 Department of Endocrinology Rigshospitalet Copenhagen University Copenhagen Denmark; 7 Department of Nuclear Medicine Pitié Salpêtrière Hospital Paris France; 8 Department of Nuclear Medicine University Hospital Marburg Marburg Germany; 9 Division of Endocrinology, Department of Medicine Helsinki University Central Hospital and University of Helsinki Helsinki Finland; 10 Division of Specific Endocrinology University Hospital Dusseldorf Dusseldorf Germany; 11 Department of Nuclear Medicine – Radioisotopic Therapy and Endocrinology Unit Instituto Nazionale Tumori Milan Italy; 12 Self-Help Organization of Thyroid Cancer Patients ‘Ohne Schilddrüse leben e.V.’ Berlin Germany; 13 Department of Endocrinology University Medical Center St Radboud Nijmegen The Netherlands; 14 Department of Nuclear Medicine RWTH University Hospital Aachen Aachen Germany

## Abstract

Differentiated thyroid cancer (DTC) is the most common endocrine cancer and its incidence has increased in recent decades. Initial treatment usually consists of total thyroidectomy followed by ablation of thyroid remnants by iodine-131. As thyroid cells are assumed to be the only source of thyroglobulin (Tg) in the human body, circulating Tg serves as a biochemical marker of persistent or recurrent disease in DTC follow-up. Currently, standard follow-up for DTC comprises Tg measurement and neck ultrasound combined, when indicated, with an additional radioiodine scan. Measurement of Tg after stimulation by endogenous or exogenous TSH is recommended by current clinical guidelines to detect occult disease with a maximum sensitivity due to the suboptimal sensitivity of older Tg assays. However, the development of new highly sensitive Tg assays with improved analytical sensitivity and precision at low concentrations now allows detection of very low Tg concentrations reflecting minimal amounts of thyroid tissue without the need for TSH stimulation. Use of these highly sensitive Tg assays has not yet been incorporated into clinical guidelines but they will, we believe, be used by physicians caring for patients with DTC. The aim of this clinical position paper is, therefore, to offer advice on the various aspects and implications of using these highly sensitive Tg assays in the clinical care of patients with DTC.

## Introduction

Differentiated thyroid carcinoma (DTC; papillary and follicular carcinoma) is the most common endocrine malignancy and is characterised by low morbidity and mortality because its clinical course is generally indolent (1). Initial treatment consists of total thyroidectomy, except in patients with microcarcinoma when lobectomy may be an option if no other nodules in the neck are detected by ultrasound (US) (2, 3). After thyroidectomy, most patients receive ablative iodine-131 (^131^I) therapy, depending on the individual patient's situation and physician preference (4). A third element of DTC treatment, levothyroxine (l-T_4_) therapy, aims to achieve either a low–normal thyrotrophin (TSH) concentration or TSH suppression, depending on the disease stage (5). With treatment, most patients with DTC have an excellent prognosis with normal life expectancy (6). However, some show persistent disease after initial therapy or develop recurrent disease during follow-up (1). Hence, life-long regular monitoring of DTC has been advocated (2, 3, 7).

Thyroglobulin (Tg) is a large glycoprotein that in healthy thyroid tissue is stored in the follicular colloid of the thyroid gland where it acts as a substrate for the synthesis of thyroid hormones. As it is produced by normal or well-differentiated malignant thyrocytes only, its tissue-specific origin makes it eminently suitable for use as a tumour marker (8). Follow-up of DTC was greatly improved by the advent of Tg measurement in the early 1980s and, due to the gradual improvements in the sensitivity and precision of Tg assays (9), the combination of Tg measurement and neck US is now regarded as the *de facto* standard of care (2, 3). However, optimal detection of smaller foci of disease has traditionally required stimulation of endogenous Tg production by high serum TSH concentrations (1, 2, 3, 10, 11).

A new generation of Tg assays has now been developed following further technological advances (12, 13) and initial reports suggest that their sensitivity is sufficient to obviate the need for TSH stimulation in patients with DTC and undetectable Tg during l-T_4_ replacement therapy (14, 15, 16, 17). This has considerable implications for clinical practice, but is not yet included in clinical guidelines (2, 3); most current clinical guidelines base their recommendations on studies performed using assays which are outdated by approximately a decade. With continuing rapid improvements in assay sensitivity, it will be important for clinicians to review the clinical performance of the assays for their population and to refine their clinical decision limits. To respond to this situation, an international group of experts convened in Zurich, Switzerland (27th September 2012), and Leiden, The Netherlands (10th September 2013), to form a consensus opinion, based on the available literature, on the implications of introducing highly sensitive Tg assays into the management of DTC. The group comprised experts in endocrinology, nuclear medicine and laboratory medicine who are involved in the care of patients with DTC, as well as representatives from thyroid cancer patient organisations.

In this paper, we report on the consensus reached during these meetings. As the volume of evidence available using these new Tg assays is still small, these statements are not intended as guidelines. However, as an increasing number of laboratories are switching to these assays, physicians will require information on their use and interpretation in DTC care. Whenever possible, physicians should refer first to the relevant guidelines, but should these not provide the required information it is our hope that this document can give some direction on how to proceed until the professional society guidelines can be updated. Until this happens, the care of patients with DTC using novel highly sensitive Tg assays will require individualised management and follow-up, both of which demand the skill and experience of a multidisciplinary team of experts in endocrinology, nuclear medicine and laboratory medicine.

## Methods

Before the consensus meeting, a review of the literature up to and including November 2013 was performed by the participants and a series of questions phrased:

1. Questions concerning analytical aspects of highly sensitive Tg assays
  1. What are the relevant analytical characteristics of Tg assays?
  2. How do we define a highly sensitive Tg assay?
  3. Which interferences with a highly sensitive Tg measurement can occur and how can they be detected?
2. Questions concerning the clinical aspects of highly sensitive Tg assays in the care of patients with DTC
  1. Indications for Tg measurement
    1. What are the current indications for Tg measurement?
    2. Does a highly sensitive Tg measurement lead to changes in the indication for Tg measurement?
    3. Can highly sensitive Tg assays be employed in patients treated with surgery alone?
  2. TSH stimulation
    1. Which patients require TSH stimulation for optimal clinical sensitivity of the Tg measurement?
    2. Can the use of highly sensitive Tg assays replace the recombinant human TSH stimulation test?
    3. When and how often should highly sensitive Tg be measured during follow-up?
    4. What are the gaps in knowledge and controversies surrounding Tg measurement and its interpretation?

**Figure 1:**
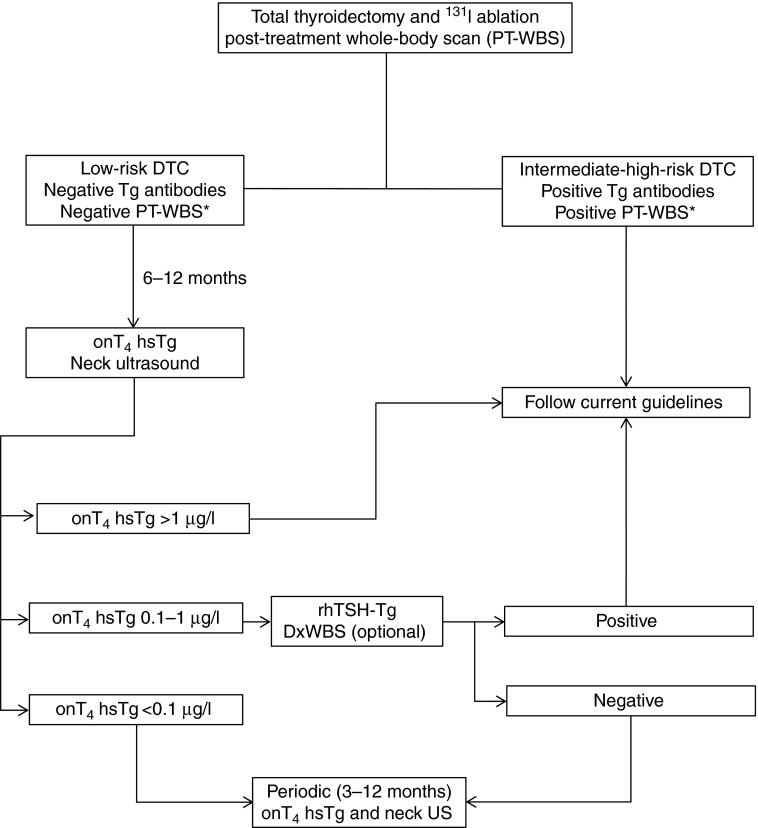
Proposed flowchart for the incorporation of highly sensitive Tg testing into routine clinical practice (note: for patients treated with surgery alone refer to ‘Can highly sensitive Tg assays be employed in patients treated with surgery alone?’ section).

During the meetings, each question and the corresponding evidence from the literature were discussed and an answer reached and affirmed by the group. Additional discussion was carried out via electronic communication where necessary. Furthermore, the available evidence was graded using the same system as for the Revised American Thyroid Association management guidelines for patients with thyroid nodules and differentiated thyroid cancer (3), which in turn was adapted from the U.S. Preventive Services Task Force, Agency for Healthcare Research and Quality (18). This grading system is summarised in Table 1. Based on the consensus opinions, a management algorithm for use in clinical practice was formulated (Fig. 1).

## Outcomes and recommendations

### 1. Questions concerning analytical aspects of highly sensitive Tg assays

#### 1.1. What are the relevant analytical characteristics of Tg assays?

The sensitivity of the assay is critical when using serum Tg measurement to detect small amounts of thyroid tissue and small changes in concentration over long time periods. Over the last few decades, immunometric assays (for example, radiometric, enzymatic and luminometric assays) have widely replaced RIAs for measuring peptides and proteins such as TG. Immunometric assays are consistently more sensitive than RIAs and, additionally, they have a shorter incubation time, wider working range and a more stable labelled antibody reagent that is less prone to labelling damage (Table 2) (8, 19, 20).

##### Analytical sensitivity

The analytical sensitivity of an assay should not be confused with the clinical sensitivity, i.e. the probability that a test will correctly identify an illness when present. Analytical sensitivity can be defined as the lowest concentration that can be reliably distinguished from 0 (8, 9, 20) and determined experimentally in a number of ways, each with advantages and limitations.

##### Functional sensitivity, limit of detection and limit of quantification

Currently, functional sensitivity is widely used to define the clinical utility of Tg assays. It is a measure of the imprecision of an assay at a low analyte concentration and involves variation due to measurement imprecision and not to biological variations. In essence, it is the variation that would be observed in many repeated measures of a single biological sample under unchanging conditions and is defined as the concentration resulting in a coefficient of variation of 20% (19).

The difference in functional sensitivity between Tg assays has created a ‘generational’ nomenclature system with each subsequent generation exhibiting a substantial improvement (i.e. tenfold) (21). To determine the functional sensitivity, the National Academy of Clinical Biochemistry (NACB) recommends assessing multiple measurements of a human serum pool (use of quality control materials is not recommended) containing a low Tg concentration over a period of 6–12 months using at least two calibrator lots and two reagent lots (21). However, this method is difficult and demanding and the data cited by the manufacturer for different Tg assays may not follow this definition. In addition, assays may be adapted over time (e.g. through reagent changes or recalibrations) even though they keep the same brand name. A good example of this is provided by the ‘high-sensitivity’ troponin assays where changes in reagent lots and calibration algorithms have led to changes in functional sensitivity (22).

The limit of detection (LOD) and limit of quantification (LOQ) are among other methods for determining the characteristics of an assay at a low analyte concentration (23, 24). LOD is the lowest analyte concentration likely to be reliably detected and distinguished from the limit of the blank, a characteristic used in assay development. LOQ is the lowest analyte concentration that can be reliably measured, with defined requirements for bias and imprecision, such as the total allowable error, often defined as ≤30%.

There are regulatory requirements for manufacturers to give details of both the analytical sensitivity of the assay and how it was determined. As functional sensitivity and LOQ are not the same, laboratories and clinicians should be aware of how the analytical sensitivity of the assay they use was assessed. Laboratories should, therefore, verify these parameters as part of their evaluation process (20, 21, 24).

**Recommendations**

- Manufacturers are recommended to report a measure of analytical sensitivity, namely the functional sensitivity and/or LOQ. *Grade B*.
- Experimental protocols adopted to define the analytical sensitivity should be reported in detail. *Grade B*.
- Laboratories should verify the analytical sensitivity for their own patient population. *Grade C*.

##### Standardisation

Tg is a large (660 kDa), highly glycosylated dimeric molecule that is heterogeneous in serum due to differential splicing of *TG* mRNA as well as carbohydrate and iodide heterogeneity. In addition, biosynthesis of the mature Tg molecule may become unregulated in thyroid tumour cells resulting in differences in the structure of the circulating TG protein. These changes can lead to exposure or masking of epitopes and hence differences in Tg immunoreactivity (19, 25). Different Tg assays employ a number of anti-Tg antibodies with varying specificity for different epitopes. Potentially, this can result in variability in the measurement of different TG isoforms in the patient's specimen and ultimately to differences in Tg concentration reported by the assays (8, 9, 20, 21).

Early international collaborative studies showed that serum Tg concentrations vary by as much as 40–60% between methods (26). The introduction and use of the Certified Reference Material (CRM 457, now described as BCR 457, European Commission, Institute for Reference Materials and Method) have significantly reduced intermethod variability to about 30%, but do not eliminate it completely (26, 27, 28). Consequently, any change in Tg assay has the potential to disrupt serial monitoring and prompt inappropriate clinical decisions. If an assay change is unavoidable, a new baseline should be established through parallel Tg measurements using both the old and the new assay (29). Furthermore, internal and external quality control programmes, including samples at low and very low Tg concentrations, are of pivotal importance for checking the precision, reproducibility (internal quality control) and accuracy (e.g. lack of bias of analytical results) of assays to ensure optimal patient care.

**Recommendations**

- Serum Tg should be measured by validated immunoassays calibrated against the Certified Reference Material (CRM 457, now described as BCR 457, European Commission, Institute for Reference Materials and Method). *Grade A*.
- Laboratories providing Tg measurement are required to participate in a certified national or international programme of quality assurance. *Grade B*.
- For longitudinal consistency of clinical care, consecutive measurements of Tg and anti-Tg autoantibody (TgAb) concentrations should be performed in the same laboratory using the same assay each time. *Grade A*.

##### Current analytical recommendations

Several general rules on how to measure Tg can be summarised from existing guidelines. All guidelines uniformly recommend performing Tg measurement using an assay calibrated against the BCR 457 standard. As there is a large variability between assays despite this standardisation, the American Thyroid Association (ATA), NACB guidelines and 2006 European Consensus on the management of thyroid cancer further recommend using the same Tg assay and the same laboratory over the course of follow-up (2, 3, 21). The European Consensus is the only guideline to state that an immunoradiometric Tg assay with a functional sensitivity <1.0 μg/l should be used. Although representing the state of the art at the time, this recommendation appears to be outdated, as newer, non-IRMAs with a better analytical sensitivity are now available. Of note, the ATA and NACB guidelines do not recommend a specific assay methodology, so any immunoassay or other techniques, such as liquid chromatography tandem mass spectrometry (30) could be used, as long as the standardisation and preferably analytical sensitivity requirements are met.

The majority of patients with DTC show an elevated pre-operative serum Tg, but the predictive role of this measurement is debatable as immunoassays cannot detect a difference between Tg from normal thyroid tissue and that secreted by thyroid cancer tissue. In clinical practice, however, patients with clear disease foci or significant thyroid remnants but undetectable serum Tg levels despite negative TgAb test results are occasionally encountered (31, 32, 33). This may suggest a total thyroidectomy, but may also occur when: the spatial conformation of Tg is changed leading to decreased immunoreactivity; the ability to secrete Tg is lost (21, 34); the TgAb result is a false negative or inappropriate cut-off values were used (35, 36, 37, 38). To overcome this problem, an ‘*in vivo*’ recovery test has been proposed: measurement of Tg and TgAb concentrations in any patient referred for thyroidectomy due to a suspicion of DTC (37, 39). This strategy could provide ‘baseline’ Tg and TgAb concentrations, which, as stated by the NACB guidelines (21), could theoretically allow assessment of the reliability of Tg and TgAb measurements after thyroidectomy, although the clinical value of this ‘*in vivo*’ test has never been verified.

**Recommendations**

- There are insufficient data in the literature to recommend any particular analytical method or assay for Tg measurement, provided that requirements for standardisation and analytical sensitivity are met. *Grade I*.
- Although measuring Tg and TgAb concentrations before thyroidectomy as differential diagnosis of a suspected or proven DTC is not recommended, a pre-thyroidectomy Tg and TgAb measurement might be used as an ‘*in vivo*’ recovery test to assess the reliability of Tg as a post-operative tumour marker. *Grade C*.

#### 1.2. How do we define a highly sensitive Tg assay?

The newest generation of Tg assays is characterised by a much improved analytical sensitivity and precision at low analyte concentrations. Using the available literature and after extensive discussion, we propose a consensus definition based on the clinical characteristics of current assays with a functional sensitivity ≤0.1 μg/l. Although assays claiming to be highly sensitive state a functional sensitivity ≤0.1 μg/l, heterogeneity in assay reactivity may lead to differences in clinical performance. Therefore, an assay with a higher functional sensitivity value when calibrated against BCR 457 (i.e. >0.1 μg/l) may have a clinical performance equal to or better than one with a functional sensitivity ≤0.1 μg/l. Details of the concepts used in this definition will be discussed later within this document.

**Recommendations**

- A highly sensitive Tg assay is defined as a Tg assay that may obviate the need for routine TSH stimulation, as its clinical sensitivity and negative predictive value for an undetectable serum Tg (as defined by the laboratory determination) without TSH stimulation are sufficient for clinical use in most patients. *Grade I*.
- Values for cut-off levels when using different highly sensitive Tg assays may differ significantly from those currently validated by clinical studies. Clinical thyroidologists and laboratory specialists are strongly advised to carefully evaluate the analytical and clinical performance of any newly introduced highly sensitive Tg assay and to derive analytical cutoffs and clinical decision limits in their own DTC patient populations. *Grade I*.

#### 1.3. Which interferences with a highly sensitive Tg measurement can occur and how can they be detected?

There is much potential for interference with Tg measurement and a considerable body of literature on the topic. Herein, we briefly summarise the most salient points and extrapolate with our opinions regarding any impact on a highly sensitive Tg measurement. More extensive information on this issue can be found in a recent position paper (39).

Depending on the population studied, the assay used and the definition of a positive test result, up to 25–30% of patients with DTC have a positive test for TgAbs at the time of diagnosis (40, 41). Hence, current guidelines and the 2006 European Consensus recommend concurrent Tg and TgAb measurements to detect any potential interference from TgAbs. There are two approaches to detect TgAbs: recovery of added exogenous Tg or measurement of TgAbs by immunoassay. While the first approach has a relatively wide reference range and is very dependent on experimental conditions (e.g. concentration of added Tg, and two measurements of Tg that increase overall imprecision), the second approach will, depending on the assay, detect most TgAb-positive patients. In this context, a positive result is often defined as a result above the upper limit of the reference range. However, due to limitations, it may be more appropriate to use a lower limit optimised for detection of analytical interference (35, 36, 37, 38) as the cut-off value for interference (37, 39).

In addition, interpretation of results is not always straightforward as not all patients with positive TgAbs will show interference by measurement of recovery or discordance between Tg results using different assays (39, 42). Any TgAb assay should be standardised against the First International Reference Preparation 65/93, but, due to the heterogeneous nature of TgAbs, no single assay can predict with absolute certainty whether TgAbs in a given sample will interfere with Tg measurement. Theoretically, a simple relationship exists between Tg and TgAbs and the higher the TgAb concentration, the higher the Tg concentration that can be concealed by TgAbs (26). Sometimes, however, seemingly low concentrations of TgAbs may be associated with strong interference and conversely patients with high concentrations of TgAbs show no evidence of interference with the Tg measurement (40). Accordingly, even low concentrations of TgAbs can, potentially, obscure the very low Tg concentrations measured by highly sensitive assays. Consequently, any assay manufacturer producing a highly sensitive Tg assay should ideally ensure that a matching highly sensitive TgAb assay is available (21).

Recently, new recovery tests using a low Tg concentration (i.e. 5–10 μg/l) have been introduced (43, 44). While the performance of these ‘low-concentration recovery tests’ has yet to be investigated extensively in patients with DTC, they may prove to serve as an addition to TgAb measurement (45).

In summary, any sample with a positive TgAb result and/or abnormal recovery test/discordant Tg result by different analytical methods (e.g. immunometric assay vs RIA) should be considered as unreliable for measuring serum Tg concentrations in patients with DTC and an alternative method is sought.

A small or moderate percentage of patients (<1–10% in the literature) show interference with Tg measurement due to heterophile antibodies (46, 47, 48). These can bind animal antigens and form a bridge between capture and detection antibody leading to a falsely elevated (or, rarely, falsely decreased) Tg measurement in immunometric assays. Heterophile antibody interference may be detected either by recovery measurement or by measurement of Tg in serially diluted sera (providing that Tg concentrations are sufficiently high) (39). An additional method, which is more specifically geared towards heterophile antibody interference, is to pretreat a serum aliquot with proprietary blocking agents and then compare the Tg result with an aliquot that was not pretreated (46, 47, 48). As with current Tg assays, one or more of these tests should be performed in patients with discordant clinical findings, such as positive imaging but undetectable Tg (using a serum highly sensitive assay) in the absence of TgAbs and/or an unusual clinical course of Tg concentrations or Tg recovery.

**Recommendations**

- For detection of serum TgAbs, a validated quantitative immunoassay standardised against the First International Reference Preparation 65/93 is recommended. *Grade C*.
- Testing for the presence of TgAbs should be performed routinely with every highly sensitive Tg measurement. The literature does not, however, offer any evidence to recommend whether or not this test should be from the same manufacturer as the Tg assay. *Grade A*.
- Conventional recovery testing for the detection of TgAb interference is not sufficiently accurate for predicting assay interference with modern highly sensitive Tg assays. *Grade D*.
- Routine screening for the presence of heterophile antibodies is not recommended; testing for heterophile antibodies or for undetected TgAbs should be performed only in patients with a dissociation between clinical and laboratory findings. *Grade C*.
- In the event of clinical suspicion of heterophile antibody interference, testing for the presence of heterophile antibodies can be performed using proprietary, commercially available heterophile antibody blocking tubes. Recovery testing and/or serial serum dilution can be considered as alternatives to the use of blocking agents. *Grade C*.
- In cases where TgAb or heterophile antibody interference is present, highly sensitive Tg measurements cannot be considered to be reliable. *Grade F*.

### 2. Questions concerning the clinical aspects of highly sensitive Tg assays in the care of patients with DTC

#### 2.1. Indications for Tg measurement

##### 2.1.1. What are the current indications for Tg measurement?

The ATA and NACB guidelines and European Consensus agree that Tg assay is an important, sensitive method for monitoring patients with DTC for the presence of residual or recurrent disease during follow-up after total thyroidectomy and adjuvant ^131^I remnant ablation. In addition, the ATA guidelines and European Consensus suggest that periodic serum Tg measurements should be considered as part of follow-up after partial thyroidectomy or total thyroidectomy without ^131^I remnant ablation. However, no interpretation criteria are mentioned as only a few studies have investigated Tg assays in the follow-up of non-ablated patients.

All guidelines and the European Consensus also agree that screening patients with thyroid nodules for the presence of thyroid cancer using Tg measurements is both insensitive and non-specific and therefore not recommended. However, Tg measurement can be employed to strengthen or exclude a suspicion of DTC in patients with widespread metastases of unknown origin, although this indication will rarely apply.

Finally, the NACB guidelines state that Tg can be used outside of DTC care to ascertain a diagnosis of thyrotoxicosis factitia, assess congenital hypothyroidism and assess the activity of inflammatory non-autoimmune thyroiditis.

##### 2.1.2. Does a highly sensitive Tg measurement lead to changes in the indication for Tg measurement?

The primary indication for Tg measurement using a highly sensitive assay will be the same as for the previous Tg assays: to monitor patients for the presence of residual or recurrent disease during DTC follow-up after initial treatment. The other indications mentioned above are also unlikely to change. Whether the interpretation of results, such as in the case of congenital hypothyroidism, will change because of the improved assay sensitivity remains to be documented.

**Recommendation**

- The use of highly sensitive Tg assays does not change the indication for Tg measurement. *Grade I*.

##### 2.1.3. Can highly sensitive Tg assays be employed in patients treated with surgery alone?

According to the various guidelines, it is sufficient to treat patients with a thyroid microcarcinoma by resection of the affected thyroid lobe only and without complete thyroidectomy or ^131^I ablation (2, 3). In this situation, measuring Tg using a highly sensitive assay, just like conventional Tg measurement, is essentially useless as Tg levels will not depend on the presence or absence of tumour foci, but rather on the remaining thyroid lobe volume, current iodine status and TSH concentration. In such patients, the options for DTC follow-up are to perform cervical US and, if recurrence or metastasis are suspected, to secure the diagnosis through a fine-needle biopsy.

In other cases, such as patients with tumours <10–20 mm, no lymph node and/or distant metastases, a (near-)total thyroidectomy without radioiodine ablation is now considered as a reasonable treatment (3, 4). These non-ablated patients may have a considerable thyroid remnant and therefore the absolute Tg concentration will be significantly less useful in this scenario. Durante *et al*. (49) explored the evolution of Tg levels over time (using an immunoradiometric Tg assay with a functional sensitivity of 0.4 μg/l) in 290 low-risk patients with DTC treated by total or near-total thyroidectomy but without ^131^I ablation and 495 matched patients treated by additional ^131^I ablation. After a median follow-up of 5 years (range: 2.5–22) the final Tg levels were <1 μg/l in 274 out of 290 non-ablated patients (95%) and 492 out of 495 ablated patients (99%). In a subgroup of 78 patients, serum Tg levels were measured serially and 47 (60%) had a serum Tg <0.4 μg/l at the first post-operative examination (3–12 months). The number of Tg-negative patients increased over time and included 79% of the group after 5 years of follow-up. In 77 cases (98.7%), Tg concentrations remained stable or declined spontaneously over time and patients remained disease free; the remaining patient was the only one to develop recurrent disease. More recently, Nascimento *et al*. (50) reported a retrospective evaluation of 86 low-risk patients with DTC treated by total thyroidectomy only (i.e. without radioiodine ablation). Serum Tg was measured using a highly sensitive assay with a functional sensitivity of 0.1 μg/l. Of the 76 patients without TgAbs, the first Tg measurement during l-T_4_ therapy and obtained at a mean time of 9 months after surgery was ≤0.1 μg/l in 62% of cases, ≤0.3 μg/l in 82%, ≤1 μg/l in 91% and ≤2 μg/l in 96% of cases. After a median follow-up of 2.5 years (range: 0.6–7.2), one patient had persistent disease, an unstimulated Tg concentration of 11 μg/l and an abnormal neck US, while two patients had Tg levels >2 μg/l with normal neck US. Within the first 2 years after total thyroidectomy the unstimulated Tg level was <0.3 μg/l in 86% and ≤2 μg/l in 96% of the cases respectively. Based on these results, the authors concluded that Tg was a useful tool to monitor patients with DTC treated by thyroidectomy alone. However, they also emphasised that the results were strictly dependent on the completeness of surgery by a dedicated surgeon in a referral centre. Overall, although a stable serum Tg concentration combined with normal neck US may be helpful in assessing whether there is concern for progressive disease, serum Tg results should be interpreted with caution taking into account both the TSH concentration and remnant thyroid volume (8). As shown by Durante *et al*., highly sensitive Tg assays are not strictly necessary in such patients. However, a lower functional sensitivity also indicates greater reliability at low levels, so, at least in theory, a rising trend in serum Tg concentration can be detected earlier and with a higher degree of precision.

**Recommendations**

- In the follow-up of patients with DTC treated by hemithyroidectomy alone, highly sensitive Tg assays will not improve the usefulness of Tg measurement. *Grade D*.
- Highly sensitive Tg assays can be used in the follow-up of patients with DTC who did not receive ^131^I ablation after total thyroidectomy, because nearly all patients without persistent or recurrent disease will have low Tg levels, which will be stable or will decline over time. *Grade C*.
- As no clear recommendations are available on how to evaluate serum Tg concentrations in patients who do not receive ^131^I ablation, their follow-up should always include careful cervical US examinations. *Grade C*.

#### 2.2. TSH stimulation

##### 2.2.1. Which patients require TSH stimulation for optimal clinical sensitivity of the Tg measurement?

Numerous guidelines explicitly exclude patients with a very low-risk stage of disease from this assessment as recurrence risk is negligible (2, 3). Patients who have not had ^131^I ablation following surgery are also excluded from TSH stimulation during follow-up as Tg will be detectable due to remaining healthy thyroid tissue and will obscure any possible tumour-related Tg level rise. Hence, the recommendation for TSH stimulation to achieve optimal Tg measurement sensitivity can be inferred to include any patient with DTC who has low- or high-risk stages of disease and received at least one course of ablative radioiodine therapy. As stated by current guidelines, stimulated serum Tg should be measured in all patients who have had remnant ablation and negative cervical US and undetectable TSH-suppressed Tg within the first year after treatment. It should be measured after T_4_ withdrawal or recombinant human TSH (rhTSH) stimulation and ∼6–12 months after ablation. An additional radioiodine whole-body scan is suggested in the follow-up of patients with high or intermediate risk of persistent disease.

Even if clear guidance on the role of a repeated stimulation test is unavailable, a ‘negative’ TSH-stimulated Tg measurement and no other evidence of recurrent disease (i.e. negative clinical examination, neck US or additional imaging procedures, when indicated) predicts a very low risk of recurrence in both low- and high-risk patients (51, 52). In these cases routine DTC follow-up should include periodic clinical examination, including neck US and Tg measurement on l-T_4_, and suppression of TSH concentration (2, 3).

##### 2.2.2. Can the use of highly sensitive Tg assays replace the recombinant rhTSH stimulation test?

Current guidelines recommend the TSH-stimulated Tg test as a main tool in the early follow-up of patients with DTC, including those with a low-risk profile. By contrast, however, studies have suggested that the additional yield of DTC recurrences was only 0.8% after rhTSH stimulation using Tg assays with a functional sensitivity of ∼1 μg/l (53, 54). In a study by Giovanella *et al*. (55), the clinical sensitivity for the detection of DTC recurrences was increased from 66 to 92% by using an assay with a functional sensitivity of 0.4 μg/l compared with 1 μg/l; specificity was unchanged. In a further study by the same group (56), unstimulated serum Tg measurement displayed a 96% negative predictive value in 117 low-risk patients with DTC, which increased to 99% when neck US was also performed. Furthermore, measuring rhTSH-stimulated Tg levels detected only one additional case of local recurrence in 104 patients with an undetectable unstimulated Tg level. Hence, the additional value of an rhTSH-stimulated Tg measurement is modest even using a non-highly sensitive assay technology, especially in low-risk patient groups.

More recently, novel highly sensitive Tg assays (with a functional sensitivity ≤0.1 μg/l) have been developed and are commercially available. A number of studies (including prospective and/or multicentre studies) were carried out to investigate the diagnostic performance of a highly sensitive Tg measurement in the follow-up of patients with DTC (14, 15, 16, 17, 57, 58, 59). Smallridge *et al*. (17) evaluated 194 patients submitted to rhTSH stimulation using the Access Tg assay (Beckmann Coulter, Fullerton, CA, USA; functional sensitivity 0.1 μg/l), and of the 80 patients with Tg <0.1 μg/l, two (2.5%) had an rhTSH-stimulated Tg >2 μg/l. None had rhTSH-stimulated radioiodine imaging suggestive of local recurrence or distant metastasis. If the unstimulated highly sensitive Tg result was 0.1–0.5 or 0.6–2.0 μg/l, the rhTSH-stimulated Tg level was >2 μg/l in 24.2 and 82.4% respectively. Schlumberger *et al*. (15) found a threshold for basal highly sensitive Tg of 0.27 μg/l for the Access Tg assay and 0.22 μg/l for the EIASON TgCa assay (Iason GmbH, Graz-Seisberg, Austria; functional sensitivity 0.02 μg/l) as below this level including a stimulated Tg measurement does not provide further information. However, this study compared unstimulated Tg levels obtained 3 months after thyroid ablation with stimulated Tg levels obtained 9–12 months later, whereas both basal and stimulated Tg levels should be measured 6–12 months after ablation (1, 2, 3). As Tg levels tend to decline continuously over time after radioiodine ablation, the data from Schlumberger *et al*. should be interpreted with caution and cannot be compared directly with those obtained in different settings. Remarkably, minimally detectable basal Tg (i.e. values between 0.1–1 μg/l) represents a 'grey zone' even if it does not necessarily predict recurrence or death (60). Malandrino *et al*. (59) found recurrent disease in 12.5% of patients with low-risk DTC when basal highly sensitive Tg levels exceeded 0.15 μg/l, while a very low risk of recurrence was found when basal highly sensitive Tg was <0.15 μg/l, even in patients with intermediate- or high-risk disease. Castagna *et al*. (58) also evaluated the clinical performance of the Access Tg and EIASON TgCa assays using a 0.1 μg/l cutoff for basal highly sensitive Tg and found that nearly 15% of disease-free patients had detectable Tg in the range of 0.1–1.0 μg/l. While 100% of these disease-free patients had a stimulated Tg <1.0 μg/l, the majority of patients (nearly 85%) with biochemical or clinically apparent disease had a stimulated Tg >1.0 μg/l. Thus, although both assays displayed a good sensitivity and an excellent negative predictive value, they showed poor specificity and positive predictive value.

The ultimate evaluation of assay performance for detecting persistent or recurrent disease is the occurrence of clinically detectable foci of disease during the course of DTC follow-up as well as biochemical detection. Zöphel *et al*. (14) evaluated 126 patients with DTC undergoing TSH-suppressive l-T_4_ therapy; at the beginning of their retrospective analyses all patients were in remission. Serum Tg was detectable (range: 0.03–0.8 μg/l) in 92 out of 116 patients (73%) using the highly sensitive EIASON TgCa method. Over the next 4 years, serum Tg concentrations remained stable in 121 (96%) patients, all of whom remained in clinical remission. In five patients serum Tg levels increased to more than double the baseline concentration and four of these five patients, showed clinically recurrent disease. Furthermore, Chindris *et al*. (61), in an extension of the aforementioned study by Smallridge *et al*. (17), reported 163 low- and high-risk patients with DTC and a basal Tg concentration <0.1 μg/l using the Access Tg assay. During a median 9.6 years' follow-up, only 4% had recurrent disease detected by US or chest X-ray. Rosario *et al*. (62) prospectively evaluated 122 patients with DTC, a negative neck US and a basal Tg concentration <0.1 μg/l as determined by the Access Tg assay 6 months after thyroidectomy and ^131^I ablation. TSH-stimulated Tg measurement was not performed. After a median follow-up of 56 months (range: 24–78), 117 patients still had no clinically apparent disease and undetectable Tg using a highly sensitive Tg assay. Serum Tg was low but detectable with a stable or decreasing trend over time in four disease-free patients and detectable following subsequent measurements in only one patient with lymph node relapse detected by neck US. In combination, the results of these studies demonstrated that TSH stimulation did not change the management of patients with an undetectable basal highly sensitive Tg assay result. Spencer *et al*. (63) have pointed out that there may be a directly proportional relationship between the basal and stimulated Tg levels measured by a highly sensitive assay. However, factors such as tumour differentiation might play an independent role in this relationship and, in addition, cutoffs for the fold rise must be confirmed in prospective clinical studies. In a systematic meta-analysis of the available literature, Giovanella *et al*. (64) demonstrated that the negative predictive value of highly sensitive Tg (<0.1 μg/l) ranged from 97 to 99%; the positive predictive value ranged from 32 to 70% when employing a stimulated Tg measurement >1 and >2 μg/l as cutoff for positivity respectively. In summary, the literature suggests that an undetectable Tg value using a highly sensitive assay (i.e. functional sensitivity <0.1 μg/l) is associated with an adequate sensitivity and negative predictive value to obviate the need for measuring TSH-stimulated Tg concentrations. However, the improved sensitivity is associated with a lower clinical specificity. Patients with a low but detectable basal highly sensitive Tg level (i.e. between 0.1 and 1 μg/l) can only be considered as ‘disease free’ after a negative TSH-stimulated Tg measurement and should therefore undergo TSH-stimulated Tg testing (64). As most patients enrolled in the available studies were affected by low-risk DTC, data on patients with intermediate- and high-risk tumours are less robust. As a result, the approach discussed should be restricted to low-risk DTC patients (65).

**Recommendations**

- A patient with low-risk DTC and an undetectable unstimulated highly sensitive Tg result will not require a stimulated Tg measurement. *Grade B*.
- Patients with an unstimulated Tg level between 0.1 and 1 μg/l measured using a currently available highly sensitive Tg assay will require at least one TSH-stimulated Tg measurement to assess their disease status. The result of this test should be interpreted in accordance with current clinical guidelines. *Grade B*.

##### 2.2.3. When and how often should highly sensitive Tg be measured during follow-up?

The guidelines are uniform in their recommendation that Tg measurements should be part of every routine follow-up examination of thyroid cancer patients. When and how often these should take place is, however, open to interpretation. The European Consensus gives no interval recommendation whereas the ATA guidelines state a frequency of every 6–12 months. In addition, it should be noted that Tg results cannot be reliably interpreted from samples collected immediately after surgery (i.e. post-surgical Tg half-life: 2–4 days) or up to 3 months after radioiodine therapy (2, 66, 67, 68). Therefore, waiting 6–8 weeks after surgery and 3 months after radioiodine therapy is recommended by the NACB guidelines and European Consensus respectively. Consequently, it is up to the attending physician to determine the exact follow-up interval within a window of 3–12 months, taking into account the patients' risk profile and the time elapsed since initial diagnosis and treatment. Given that patients with an undetectable basal highly sensitive Tg result are at low risk of recurrence, but do not have any risk, it appears sensible to measure highly sensitive Tg at every routine follow-up examination allowing for suitable time intervals after medical interventions that may cause an elevation in serum Tg levels.

**Recommendations**

- Physicians should wait a minimum of 6 weeks after surgery and 3 months after ^131^I therapy to measure Tg; should a prior measurement nonetheless take place, this should be interpreted with extreme caution. *Grade C*.
- Tg measurement without TSH stimulation should be performed at 3–12 month intervals; the exact frequency needs to be determined for each patient taking into account the stage and the course of disease. *Grade C*.

##### 2.2.4. What are the gaps in knowledge and controversies surrounding Tg measurement and its interpretation?

The main controversy in the current guidelines is the definition of a ‘disease-free’ patient. Diagnostic criteria for successful ablation and the absence of persistent disease are a serum Tg concentration <1 μg/l (European Consensus) or <2 μg/l (ATA guidelines) following TSH stimulation and a normal neck US. Diagnostic ^131^I whole-body scanning is recommended for intermediate- and high-risk patients, in all patients with positive TgAbs and/or a positive post-ablation scan (i.e. a scan performed a few days after ^131^I therapy administration) and in those with a positive stimulated Tg but negative neck US (3). Standardising the reporting criteria for US and radioiodine diagnostic scanning will help to better define a disease-free status in combination with Tg measurements (69). However, the cut-off levels reported in the literature and guidelines for basal and stimulated Tg are predominately based on assays with a functional sensitivity of 0.5–2 μg/l. In addition, recent evidence has suggested that TSH-stimulated Tg levels depend, in part, on the duration and level of stimulation (70), implying that any cutoff is effectively meaningless if different stimulation methods and intervals are used. Furthermore, the guidelines do not take a clear stance on patients with Tg concentrations between the functional sensitivity and the recommended cutoff, as measured by the newer assays. In light of the uncertainty pertaining to the meaning of such low detectable Tg levels, some authors prefer to use the functional sensitivity or the LOQ of the Tg assay to define a disease-free patient after TSH stimulation (71). A further relevant point of discussion is that most guidelines do not sufficiently address which variable to use as the lower reporting limit of a particular assay – functional sensitivity or LOQ – although previously the functional sensitivity appears to have been the accepted norm.

## Conclusions

Post-surgical follow-up of DTC aims to identify early the small proportion of patients with residual disease or who will develop recurrence. In the absence of TgAbs and heterophile antibodies, Tg measurement is the reference standard for clinical management of patients previously treated for DTC. As current clinical guidelines are still based on study results obtained using Tg assays with a functional sensitivity of 1–2 μg/l, they also mandate the use of a TSH-stimulated Tg measurement to achieve sufficient clinical sensitivity for detecting persistent and/or recurrent disease. However, following the introduction of highly sensitive Tg assays, there is an increasing body of evidence that an undetectable highly sensitive Tg measurement during l-T_4_ treatment is sufficient to forgo TSH stimulation in low-risk patients with DTC. Patients with a slightly increased basal highly sensitive Tg concentration (e.g. 0.1–1 μg/l) should undergo a TSH-stimulated Tg measurement. It should also be noted that some challenges exist with these assays, especially with regard to the sensitivity for detecting interfering autoantibodies against Tg. Based on our conclusions, we formulated a modified DTC management algorithm that includes highly sensitive Tg assays and is shown in Fig. 1.

## Figures and Tables

**Table 1:** Rating and definition of the recommendations based on available evidence; definitions are identical to those used by Cooper *et al*. (3) in the American Thyroid Association Guidelines.

**Rating**	**Definition**
A	Strongly recommends. The recommendation is based on good evidence that the service or intervention can improve important health outcomes. Evidence includes consistent results from well-designed, well-conducted studies in representative populations that directly assess effects on health outcomes
B	Recommends. The recommendation is based on fair evidence that the service or intervention can improve important health outcomes. The evidence is sufficient to determine effects on health outcomes, but the strength of the evidence is limited by the number, quality or consistency of the individual studies, generalisability to routine practice; or indirect nature of the evidence on health outcomes
C	Recommends. The recommendation is based on expert opinion
D	Recommends against. The recommendation is based on expert opinion
E	Recommends against. The recommendation is based on fair evidence that the service or intervention does not improve important health outcomes or that harms outweigh benefits
F	Strongly recommends against. The recommendation is based on good evidence that the service or intervention does not improve important health outcomes or that harms outweigh benefits
I	Recommends neither for nor against. The panel concludes that the evidence is insufficient to recommend for or against providing the service or intervention because evidence is lacking that the service or intervention improves important health outcomes, the evidence is of poor quality or the evidence is conflicting. As a result, the balance of benefits and harms cannot be determined

**Table 2:** Technical and analytical characteristics of different highly sensitive Tg immunoassays.

**Assay**	**Manufacturer**	**Method**	**Standardisation BCR 457**	**Analytical sensitivity** (manufacturers data)			**Analytical sensitivity** (literature data)			
LOD	LOQ	FS	LOD	LOQ	FS	Reference
Access Tg	Beckman Coulter (USA)	ICMA	Yes	0.1^a^	NQ	NQ	0.01	NQ	0.1	(13)
EIASON TgCa	Iason (Austria)	ELISA	Yes	0.01	NQ	0.02	0.01	NQ	0.02	(12)
ELECSYS Tg II	Roche	ECLIA	Yes	0.04	0.1	NQ	NA	NA	NA	NA
KRYPTOR usTg	BRAHMS (Germany)	TRACE	Yes	0.09	NQ	0.15	NA	NA	NA	NA

ECLIA, electrochemiluminescence immunoassay; FS, functional sensitivity; ICMA, immunochemiluminometric assay; LOD, limit of detection; LOQ, limit of quantification; NA, not available; NQ, not quoted; TRACE, time-resolved amplified cryptate emission.

a Package insert reports the term ‘analytical sensitivity’.
